# Study of antimicrobial potential and cytotoxic of *Cordia nodosa *species

**DOI:** 10.1186/1753-6561-8-S4-P69

**Published:** 2014-10-01

**Authors:** Raíssa Fernanda Evangelista Pires dos Santos, Isabelle Souza de Mélo Silva Silva, Lukas Reis d'Costa, Andriele Mendonça Barbosa, Klebson Santos Silva, Mariene Ribeiro Amorim, Fernando Mendonça Diz, Thais Honório Lins, Regina Célia Santos Sales Verissimo, Francine Ferreira Padilha, Eliane Aparecida Campesatto, Maria Lysete de Assis Bastos

**Affiliations:** 1 Laboratório de Pesquisa em Tratamento de Feridas, Universidade Federal de Alagoas, Campus A.C. Simões - Av. Lourival Melo Mota, s/n, Cidade Universitária - Maceió, Alagoas, 57072-900, Brazil; 2 Instituto de Tecnologia e Pesquisa, Universidade Tiradentes - Av. Murilo Dantas, Farolândia - Aracaju, Sergipe, 49032-490, Brazil; 3 Laboratório de Farmacologia e Imunidade, Universidade Federal de Alagoas, Campus A. C. Simões - Av. Lourival Melo Mota, s/n, Cidade Universitária - Maceió, Alagoas, Brasil

## Background

Increasingly infectious agents change, which is why there is a failure in the treatment of microbial infections, above all, the emergence of resistance to existing antibiotics [1]. Therefore, the search for new antimicrobial substances from natural products has increased the interest of researchers. Brazil is considered the country has one of the greatest biodiversity on the planet, where the popular use of medicinal plants has become an important source for discovery of new active compounds [2]. However, for the safe use of these plant species, popular knowledge must relate to the performance of bioassays that demonstrate the therapeutic efficacy and low toxicity. Cordia species belonging to the family Boraginaceae, are used in folk medicine to treat gastric ulcers and proven front possess antimicrobial potential *Escherichia coli *and *Staphylococcus aureus *[3]. Phytochemical studies carried out with species of this family revealed the presence of alkaloids, naphthoquinones, saponins, tannins, flavonoids, among others, responsible for this spectrum of biological activities. *Cordia nodosa *specie has proven antifungal activity but the work as they relate to the antibacterial activity are scarce [4]. The aim of this study was to evaluate the antimicrobial activity and toxicity of *Cordia nodosa*.

## Methods

The experimental research was conducted in the Research Laboratory of Wound Care, Federal University of Alagoas. Four fractions were tested in different parts of *Cordia nodosa*, A, B, C and D. Antimicrobial activity was determined by microbial sensitivity tests, the method of disk diffusion (DD) and broth microdilution method for determination of minimum inhibitory concentration (MIC). The percentage inhibition of bacterial disk diffusion test was calculated by dividing the average inhibition hundred times the sample by the average zone of inhibition positive control [5]. 8 were used bacterial strains, among them Gram-positive and Gram-negative bacteria, distributed by American Type Cell Collection. The toxicity evaluation of the samples was measured by testing toxicity on *Artemia salina*.

## Results and conclusions

Samples B and C were considered moderately active against the strain of *Staphylococcus aureus*, with a percent inhibition of 29.2% (inhibition = 8 mm) and 55.6% (inhibition = 15 mm), respectively, when compared with the mean of the positive control used Gentamicin (about 27 mm). Both samples were also moderately active against the strains of *S. epidermidis*, involving inhibition percentages of 33.4%. The sample C presented little active front of the line of *P. aeruginosa *as the percentage of inhibition was less than 25%. The results obtained with the MIC determination showed that the sample B is the fraction of better antimicrobial activity inhibiting the growth of the strain of *S. epidermidis *concentration of 1000 to 125 mg mL^- 1^. Identified the absence of toxicity in all samples, because the mortality of *Artemia salina *was ≤ 30%. This finding dismissed the test quantitative, and therefore the LC_50 _determination, because it means that the concentrations tested were below the LC_50 _of each sample (LC_50 _≥ 1000 mg mL^-1^). These results represent the first evidence of safety of plant species that are performed in sequence *in vivo* bioassays.
